# Radiate and Planar Multipolar Neurons of the Mouse Anteroventral Cochlear Nucleus: Intrinsic Excitability and Characterization of their Auditory Nerve Input

**DOI:** 10.3389/fncir.2017.00077

**Published:** 2017-10-18

**Authors:** Ruili Xie, Paul B. Manis

**Affiliations:** ^1^Department of Neurosciences, University of Toledo, Toledo, OH, United States; ^2^Department of Otolaryngology/Head and Neck Surgery, University of North Carolina at Chapel Hill, Chapel Hill, NC, United States; ^3^Department of Cell Biology and Physiology, University of North Carolina at Chapel Hill, Chapel Hill, NC, United States

**Keywords:** cochlear nucleus, inhibition, stellate neuron, radiate, planar, auditory nerve input, excitatory synaptic transmission, synaptic dynamics

## Abstract

Radiate and planar neurons are the two major types of multipolar neurons in the ventral cochlear nucleus (VCN). Both cell types receive monosynaptic excitatory synaptic inputs from the auditory nerve, but have different responses to sound and project to different target regions and cells. Although the intrinsic physiology and synaptic inputs to planar neurons have been previously characterized, the radiate neurons are less common and have not been as well studied. We studied both types of multipolar neurons and characterized their properties including intrinsic excitability, synaptic dynamics of their auditory nerve inputs, as well as their neural firing properties to auditory nerve stimulation. Radiate neurons had a faster member time constant and higher threshold current to fire spikes than planar neurons, but the maximal firing rate is the same for both cell types upon large current injections. Compared to planar neurons, radiate neurons showed spontaneous postsynaptic currents with smaller size, and slower but variable kinetics. Auditory nerve stimulation progressively recruited synaptic inputs that were smaller and slower in radiate neurons, over a broader range of stimulus strength. Synaptic inputs to radiate neurons showed less depression than planar neurons during low rates of repetitive activity, but the synaptic depression at higher rates was similar between two cell types. However, due to the slow kinetics of the synaptic inputs, synaptic transmission in radiate neurons showed prominent temporal summation that contributed to greater synaptic depolarization and a higher firing rate for repetitive auditory nerve stimulation at high rates. Taken together, these results show that radiate multipolar neurons integrate a large number of weak synaptic inputs over a broad dynamic range, and have intrinsic and synaptic properties that are distinct from planar multipolar neurons. These properties enable radiate neurons to generate powerful inhibitory inputs to target neurons during high levels of afferent activity. Such robust inhibition is expected to dynamically modulate the excitability of many cell types in the cochlear nuclear complex.

## Introduction

The cochlear nuclear complex contains a variety of neurons that are thought to process different aspects of acoustic environment. Among these are two types of multipolar neurons in the ventral cochlear nucleus (VCN), termed planar and radiate neurons, according to the orientation of their dendrites relative to the fascicles of the auditory nerve (Doucet and Ryugo, 1997). The dendrites of the planar neurons run parallel to the sheets of auditory nerve fibers, and are thus aligned to receive input from spiral ganglion cells (SGCs) that in turn receive input from narrow frequency region of the cochlea. In contrast, radiate neuron dendrites frequently run across the auditory nerve fiber bundles, and can receive inputs from SGCs innervating a broad frequency region of the cochlea. The planar neurons are often called T-stellate cells, because their axons project out of the cochlear nucleus through the trapezoid body (Oertel et al., 1990). These cells also correspond to the “type I” multipolar neurons with sparse somatic synaptic innervation (Cant, 1981; Smith and Rhode, 1989). In contrast, the radiate neurons are also called D-stellate cells because their axons project dorsally to the dorsal cochlear nucleus (DCN; Oertel et al., 1990). They correspond to “type II” multipolar neurons that have numerous somatic synaptic contacts (Cant, 1981; Smith and Rhode, 1989). The anatomical and physiological features of both cell types have been widely studied *in vivo* (Smith and Rhode, 1989; Winter and Palmer, 1995; Palmer et al., 1996, 2003; Arnott et al., 2004; Smith et al., 2005; Needham and Paolini, 2006). While the planar multipolar cells have been previously studied *in vitro* (Oertel et al., 1990, 2011; Cao and Oertel, 2010), the radiate multipolar cells are not as numerous, and at least in mouse, appear to be unevenly distributed in the VCN. As a result, less is known about their intrinsic excitability and the dynamics of synaptic inputs from the auditory nerve.

Planar and radiate multipolar neurons respond differently to sound and serve distinct functions for auditory information processing. In response to best frequency tone bursts, planar neurons fire action potentials with regular inter spike intervals (chopping response) through the duration of a tonal stimulus and give rise to a peristimulus time histogram (PSTH) called a sustained (chop-S) or transient chopper (chop-T; Rhode et al., 1983; Rouiller and Ryugo, 1984; Blackburn and Sachs, 1989; Paolini and Clark, 1999; Paolini et al., 2005). In contrast, radiate neurons fire briefly with a regular inter spike interval at the onset of a tonal stimulus, followed by less synchronized sustained firing. The resulting PSTH is called onset chopper (O_c_; Rhode and Smith, 1986; Smith and Rhode, 1989; Winter and Palmer, 1995; Palmer et al., 1996, 2003; Smith et al., 2005). Planar multipolar cells are excitatory (Smith and Rhode, 1989; Doucet et al., 1999), and form one of the major ascending auditory projections that innervate both the DCN (Oertel et al., 1990, 2011; Doucet et al., 1999) and the inferior colliculus (Cant, 1982; Adams, 1983). Planar multipolar cells are narrowly tuned individually to sound frequency, but are also sensitive to the temporal envelopes of sounds (Rhode and Smith, 1986; Blackburn and Sachs, 1990; Frisina et al., 1990; Rhode and Greenberg, 1994), which are an important cue used in speech discrimination (Shannon et al., 1995; Swaminathan and Heinz, 2012). Radiate neurons, on the other hand, are glycinergic inhibitory neurons (Cant, 1982; Wenthold, 1987; Wickesberg et al., 1994; Doucet et al., 1999; Doucet and Ryugo, 2006). They respond strongly to broadband noise as well as tones, and project to neighboring neurons within the VCN (Smith and Rhode, 1989; Jiang et al., 1996; Palmer et al., 1996; Arnott et al., 2004; Campagnola et al., 2014), to the ipsilateral DCN (Rhode et al., 1983; Oertel et al., 1990), and via a commissural pathway to the contralateral cochlear nucleus (Needham and Paolini, 2003; Arnott et al., 2004; Smith et al., 2005). The broadband inhibition from radiate neurons has been proposed to improve the temporal representation of sounds in planar neurons (Xie and Manis, 2013b), promote the detection of signals in noise (Pressnitzer et al., 2001), and help to shape the distinct spectral response maps of DCN pyramidal cells (Nelken and Young, 1994).

As a major cell type in the VCN, planar neurons have been extensively studied *in vitro*, where their intrinsic membrane properties and the dynamics of excitatory and inhibitory synaptic inputs have been well characterized (Ferragamo et al., 1998; McGinley and Oertel, 2006; Cao and Oertel, 2010; Oertel et al., 2011; Xie and Manis, 2013b). Radiate neurons, which are fewer in number (Doucet and Ryugo, 1997), have been less extensively investigated *in vitro*. Fujino and Oertel (2001) showed that planar and radiate multipolar neurons have similar resting membrane potentials and firing patterns, and that planar cells have a smaller and slower hyperpolarization-activated cation current (I_h_) than radiate cells. Planar neurons, but not radiate neurons, are excited by activation of both nicotinic and muscarinic cholinergic receptors. The cholinergic innervation of the VCN arises (in rat) largely but not exclusively from the ventral nucleus of the trapezoid body (Sherriff and Henderson, 1994; Mellott et al., 2011). Although both planar and radiate neurons receive excitatory input from the auditory nerve, the relative strengths and dynamics of this input are not known.

In the present study, we examined the intrinsic excitability, the synaptic properties of the excitatory auditory nerve inputs, and auditory nerve evoked firing properties in both radiate and planar multipolar neurons. We found that radiate neurons have higher current thresholds and are overall less excitable than planar neurons. As a result, they respond only to stronger synaptic inputs, and may therefore provide inhibition to target neurons only during louder or wideband sounds. In addition, the spontaneous and evoked excitatory synaptic inputs in radiate neurons are significantly smaller in amplitude, and show slower but variable kinetics than those in planar neurons. The weaker synaptic inputs onto radiate neurons are in part offset by a higher synaptic convergence and slower EPSC kinetics. The slower EPSC kinetics increases temporal summation of synaptic inputs. The synaptic inputs also show less synaptic depression during repetitive stimulation. Consequently, the synaptic currents onto radiate neurons carry significantly more charge during repetitive stimulation than in planar cells. These features are consistent with the proposition that radiate neurons integrate a large number of weak inputs from SGCs over a range of characteristic frequencies, to provide sustained and robust inhibition to target neurons.

## Materials and Methods

Experiments were performed following protocols approved by the Institutional Animal Care and Use Committees at the University of North Carolina at Chapel Hill and the University of Toledo.

### Animals

Sixty-five CBA/CaJ mice between 27–44 days old and of either sex were used. Mice were purchased from Jackson Labs (Bar Harbor, ME, USA) to create breeding colonies, and maintained in animal facilities at the University of North Carolina at Chapel Hill and the University of Toledo.

### Brain Slice Preparation

All recordings were made from parasagittal brain slices containing the cochlear nucleus as previously described (Xie and Manis, 2013a,b, 2017; Xie, 2016). Mice were anesthetized with an intraperitoneal injection of ketamine (100 mg/kg) and xylazine (10 mg/kg), decapitated, the brainstem removed from the skull and immersed into artificial cerebrospinal fluid (ACSF). The standard ACSF contained (in mM): 122 NaCl, 3 KCl, 1.25 NaH_2_PO_4_, 25 NaHCO_3_, 20 glucose, 3 myo-inositol, 2 sodium pyruvate, 0.4 ascorbic acid, 2.5 CaCl_2_, and 1.5 MgSO_4_. ACSF with divalent ion concentrations close to those in normal cereberospinal fluid was also used for some recordings. This solution contained (in mM): 122 NaCl, 3 KCl, 1.25 NaH_2_PO_4_, 25 NaHCO_3_, 20 glucose, 3 myo-inositol, 2 sodium pyruvate, 0.4 ascorbic acid, 1.2 CaCl_2_, and 0.8 MgSO_4_. ACSF was warmed to 34°C and gassed with 5% CO_2_ and 95% O_2_. The brainstem was divided into two halves at the midline and one parasagittal slice was cut from each side. The brain slices were 350 μm in thickness and contained all three regions of the cochlear nucleus, the anteroventral, posteroventral and dorsal cochlear nucleus (AVCN, PVCN and DCN, respectively). Slices were incubated in ACSF at 34°C for at least 30 min to allow cells to recover from the slicing procedure before attempting recordings.

### Electrophysiological Recordings

Brain slices were transferred to the recording chamber, where they were bathed in a continuous flow (~3 ml/min) of ACSF at 34°C. Cells were visualized using 40× water immersion objective on a fixed-stage microscope (Axioskop FS2+ or Axio Examiner, Zeiss, Germany). Images were acquired with a camera (Retiga 2000DC or optiMos sCMOS, QImaging, Vancouver, BC, Canada). Whole-cell patch clamp recordings were made using pipettes pulled from borosilicate glass (KG-33; King Precision Glass, Claremont, CA, USA) with a Sutter P2000 Puller (Sutter Instruments, San Francisco, CA, USA). For current clamp recordings, the pipette solution contained (in mM): 126 K-gluconate, 6 KCl, 2 NaCl, 10 HEPES, 0.2 EGTA, 4 Mg-ATP, 0.3 Tris-GTP, and 10 Tris-phosphocreatine, with pH adjusted to 7.2 with KOH. For voltage clamp recordings, the pipette solution contained (in mM): 105 CsMetSO_3_, 35 CsCl, 5 EGTA, 10 HEPES, 4 MgATP, 0.3 Tris-GTP, 10 Tris-phosphocreatine, and 3 QX-314 (chloride salt), with pH adjusted to 7.2 with CsOH. Junction potentials were calculated to be −12 mV for K-gluconate based pipette solution and −7 mV for the Cs-based pipette solution. All reported voltages have been corrected for the appropriate junction potentials. Recordings were made with a Multiclamp 700B amplifier (Molecular Devices, Sunnyvale, CA, USA) controlled by a MATLAB program or pClamp software. In voltage clamp experiments, cells were held at −77 mV, and series resistance was compensated by at least 75% online. Excitatory inputs were evoked by electrically stimulating the auditory nerve root with a 75 μm diameter concentric electrode (FHC, Bowdoin, ME, USA), with the stimulating currents ranging between 0 μA and 400 μA. No drugs were used in the current clamp recordings. In all voltage clamp recordings, strychnine (2 μM) was used to block glycinergic inhibitory currents and isolate EPSCs. In a subset of cells, SR95531 (10 μM) was also used to block GABAergic inhibition. However, as we have observed previously (Xie and Manis, 2014), GABAergic inhibition contributes to only a very small fraction of the inhibitory currents in AVCN in slices, and was negligible at the holding voltage of −77mV in this study, regardless of whether SR95531 was applied or not. All chemicals were purchased from Sigma-Aldrich except SR95531 and CNQX, which were ordered from Tocris Bioscience (Bristol, UK).

### Cell Identification

**Figure 1:**
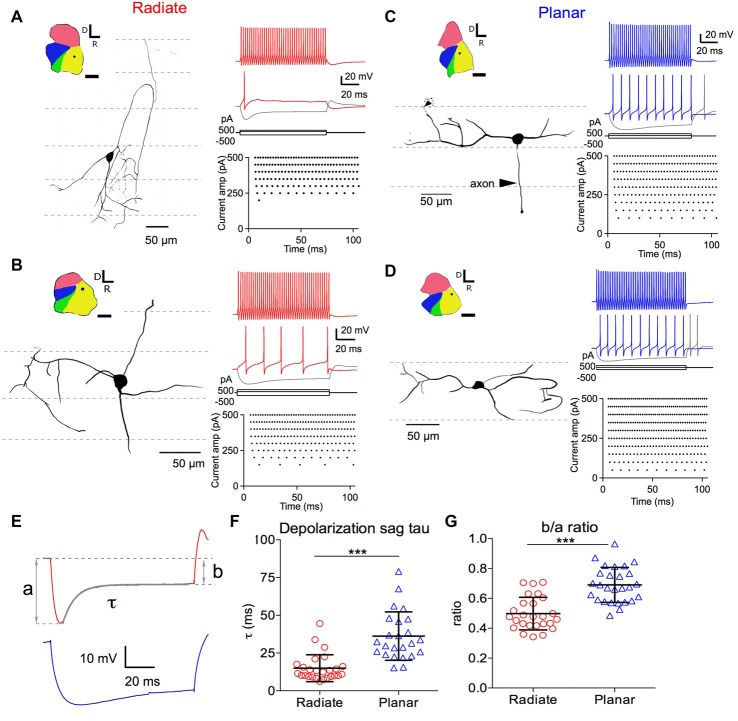
Morphology of radiate and planar neurons and current-evoked responses. Two example radiate neurons **(A,B)** and two example planar neurons **(C,D)** are shown. **(A)** Morphology and current evoked responses of an example radiate cell. Left panel: drawing of the cell morphology (see “Materials and Methods” section). Dashed lines mark the direction of auditory nerve fibers in the anteroventral cochlear nucleus (AVCN), corresponding to the iso-frequency sheets. Inset shows the diagram of the cochlear nucleus slice with the location of the neuron marked as the dot in AVCN. Regions of the cochlear nucleus are color coded to show the auditory nerve root area in green, AVCN in yellow, PVCN in blue, and dorsal cochlear nucleus (DCN) in red. All cochlear nuclei are shown in the same orientation, although recordings were made in slices from both left and right side cochlear nuclei. Calibration bar: 500 μm. Right top panel: responses to current injections at three levels: +500 pA, 200 pA (threshold current level), and −500 pA (trace in gray). Right bottom panel: raster plot of spikes evoked at different current injection levels (Current amp). Notice that only a single onset spike was evoked at the threshold current of 200 pA. **(B–D)** Example neurons with the same layout as in **(A)**. Notice that radiate neurons have dendrites that extend across the auditory nerve fibers **(A,B)**, whereas planar neurons have dendrites that run parallel to the auditory nerve fibers **(C,D)**. Current levels in all neurons included +500 pA (color) and −500 pA (gray), and a just- suprathreshold depolarizing current (in color; current levels are +150 pA in **(B)**, +100 pA in **(C)**, +50 pA in **D**). **(E)** Quantification of the depolarizating sag using responses to 100 ms current injections that hyperpolarized the membrane potential to a peak of −90 mV. Example traces are from the radiate neuron in **(A)** (red) and the planar neuron in **(C)** (blue), respectively. a: peak hyperpolarizing potential; b: steady state hyperpolarizing potential at the end of the 100 ms current injection; τ: decay time constant of the depolarization sag by fitting an exponential curve from the peak hyperpolarization to the end of current injection, as shown by the overlay in gray. **(F)** Summary of τ for both cell types. **(G)** Size of the depolarization sag quantified as b/a ratio. ***p < 0.001.

Recordings were made from cells in the dorsal half of the AVCN, corresponding to the high frequency region (Muniak et al., 2013). Cells were identified by their morphology as visualized by including 0.1% Alex Fluor 488 (Molecular Probes, Eugene, OR, USA) in the pipette solution and examining the fluorescence as excited by a 470 nm LED through a standard fluorescein filter set. Bushy cells were identified by having one or two short primary dendrites with heavily branched distal tufts (Cant and Morest, 1979; Tolbert et al., 1982; Webster and Trune, 1982; Rouiller and Ryugo, 1984; Wu and Oertel, 1984; Lauer et al., 2013), and were excluded from this study. Planar and radiate multipolar neurons differ from bushy neurons by having thin and long dendrites without a profusely branched tuft. Planar and radiate neurons were distinguished from each other by the primary orientation of their dendrites relative to the fascicles of auditory nerve fibers. Dendrites of the planar neurons are oriented primarily parallel to the fascicles of auditory nerve fibers, whereas radiate neurons had dendrites that crossed multiple fascicles (Doucet and Ryugo, 1997; Figure 1). Neurons with morphology that could not unequivocally be assigned as radiate and planar classes (for example, because of incompletely filled dendrites) were not included in the analysis. Drawings of the neural morphology were made by tracing the cells from stacks of fluorescent images taken *in situ* at the end of physiological recordings, using GIMP (version 2.8.4)^1^.

### Data Analysis

Current-firing rate curves were fit to a piecewise function consisting of linear and exponential phases:

(1) $$\begin{array}{c} for\;I\;<\;I_{\text{break}}:F(I)\;=\;F_{0}\;+\;F_{\text{b}}*\;I/I_{\text{break}}\\ for\;I\;>\;=\;I_{\text{break}}:F(I)\;=\;F_{\text{b}}\;+\;F_{\max}\;*\;(1.0\;-\;e^{-(I-I_{\text{break}})/I_{\text{r}}}) \end{array}$$

where *I* is the current; *I*_break_ defines the point where the firing rate changes from a linear function of current to the exponential, *F*_0_ is the firing rate at zero current, *F*_b_ is the firing rate at the break point, *F*_max_ is the maximal firing rate (extrapolated), and *I*_r_ is the rate at which firing increases with current during the exponential phase. Each cell was individually fit, using a sequential least squares algorithm (SLSQP) with bounds from scipy.optimize^2^, and parameters were compared across each population. Cells that showed a decrease in firing rate with increasing current of more than 25% of their maximal rates were classified as exhibiting depolarization block, and were excluded from the fitting (3 of 55 cells).

Spontaneous excitatory postsynaptic current (sEPSCs) events in radiate neurons had highly variable decay time courses, making it difficult to detect and measure sEPSCs using the scaled-template method (Clements and Bekkers, 1997) as in our previous studies (Rich et al., 2010; Xie and Manis, 2013a,b). Instead, sEPSCs were visually identified and analyzed in MiniAnalysis (Synaptosoft, Fort Lee, NJ, USA). This approach was effective for detecting events with a range of kinetics, and allowed the inclusion of smaller events that would otherwise be missed using the fixed criterion of the scaled-template method. The sEPSC rising phase was characterized by measuring the time it takes for the sEPSC to rise from 10% to 90% of its peak amplitude. The time course of the sEPSC falling phase was measured as the time to decay to 37% of the peak amplitude (37% decay time). Decay time constants were not determined from exponential fits to the sEPSCs because the small, noisy and highly variable sEPSCs could not be consistently fit. The same methods were used to measure sEPSCs in all radiate and planar neurons.

All other analyses were done in Igor Pro (WaveMetrics, Portland, OR, USA). Membrane input resistance was calculated as the slope of the current-voltage relationship from responses to small hyperpolarizing current injections. The membrane time constant was estimated by fitting the responses to small hyperpolarizing current injections from onset to the negative peak with single exponential curves. We measured the vector strength (Goldberg and Brown, 1969; Xie, 2016) of the spike times in response to trains of auditory nerve stimulation to quantify the ability of these two types of cells to encode temporal information. The stimulus trains consisted of 50 regularly-spaced constant-current shocks at 50, 100, 200 and 400 Hz.

### Statistical Analysis

Statistical analyses were performed using GraphPad Prism (GraphPad Software Version 6.0h, San Diego, CA, USA) and R (version 3.4.0, The R Foundation). Data were first tested to see if they were normal distributed using D’Agostino and Pearson omnibus normality test. If the distributions passed the normality test, then a Student’s *t*-test, a one-way ANOVA or a two-way ANOVA were used as appropriate. If the data did not follow a normal distribution, non-parametric tests (Mann-Whitney or Kruskal-Wallis test) were used. When significant main effects were indicated by an ANOVA, multiple comparison tests (Dunn’s, Tukey’s or Bonferroni corrected test as indicated in the text) were also performed. Linear mixed models with repeated measures were used to compare datasets when there were missing samples for some measurements (ANOVAs cannot handle missing observations). Linear mixed model calculations were performed in R using the package *lme4* (V1.1-13); F-tests between models were evaluated with *pbkrtest* (version 0.4-7), and post-tests were done using *glht* from the *multcomp* package (version 1.4-6) with the *p*-values adjusted for multiple comparisons using Holm’s method. Data are presented as mean ± standard deviation.

## Results

### Two Types of Multipolar Cells in AVCN Have Different Intrinsic Membrane Properties

We identified the radiate and planar multipolar neurons in AVCN morphologically, according to the orientation of their dendritic trees (Doucet and Ryugo, 1997), and evaluated the intrinsic membrane properties of these neurons by studying their responses to intracellularly injected current steps. Example cells from each group are shown in Figure 1. Cells with dendrites that left the cell body in directions that bore no relationship to the fascicles of auditory nerve fibers (Figures 1A,B; path of auditory nerve fibers shown with dotted lines) were classified as radiate cells. In contrast, cells with dendrites that were predominantly aligned with the fascicles of auditory nerve fibers were classified as planar cells (Figures 1C,D). The morphological identification of cell types was further validated physiologically in response to hyperpolarizing current injections as shown in Figures 1E–G, in which radiate multipolar neurons showed significantly faster and larger depolarization sags than planar multipolar neurons, consistent with previous reports (Fujino and Oertel, 2001; Rodrigues and Oertel, 2006). The time constant (τ) of the depolarizing sag to 100 ms current injections that hyperpolarized the membrane potential to a peak value of −90 ± 2 mV was measured by fitting a single exponential curve from the peak of the hyperpolarization to the end of the current pulse. The time constants were 15.0 ± 8.9 ms (*n* = 26) in radiate cells and 36.23 ± 16.0 ms, *n* = 24) in planar cells (Figures 1E,F; Mann Whitney test: *p* < 0.0001). The relative amplitude of the sag was determined from the ratio of the steady-state and the peak voltage (b/a ratio; Figure 1E). The b/a ratio was significantly smaller in radiate cells than in planar cells (radiate: b/a = 0.50 ± 0.11, *n* = 26; planar: b/a = 0.69 ± 0.12, *n* = 27; Figure 1G; unpaired *t* test: *t*_(51)_ = 6.17, *p* < 0.0001), suggesting that I_h_ currents are more prominent in radiate cells. The previously reported difference in spike shapes, in which radiate neurons show double undershoots after every action potential and planar neurons only show a single undershoot (Fujino and Oertel, 2001; Rodrigues and Oertel, 2006), was not obvious in this study. The disparity may be due to the differences in the ion concentrations of the electrode solutions between the present and the previous studies, as well as the difference in recording sites. In the present study all neurons recorded were from the AVCN, whereas the previous studies recorded mostly from PVCN.

**Figure 2:**
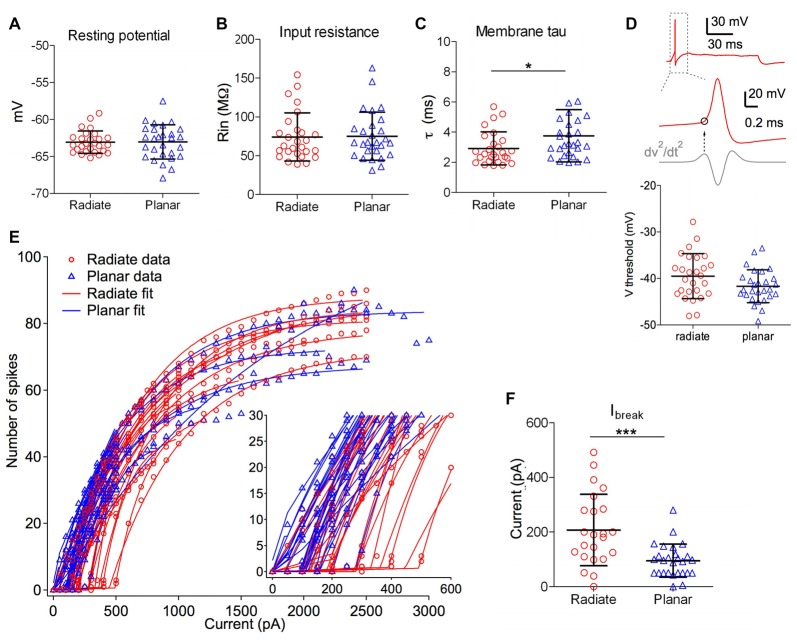
Intrinsic membrane properties of radiate and planar cells. **(A–C)** Summary plot of resting membrane potential **(A)**, membrane input resistance **(B)**, and membrane time constant **(C)** from both cell types. **(D)** Summary plot of the threshold potential (V_threshold_) from the first action potential evoked by minimum current injections (bottom panel). Top panel: example trace from a radiate neuron with an action potential evoked by the minimum current injection level. Middle panel: V_threshold_ (marked as the circle) was measured as the membrane potential at which the second derivative (dV^2^/dt^2^) was maximal during the rising phase of the action potential. **(E)** Current-spike relationship for all radiate and planar cells. Each line represents an individual cell. Points: data; lines: best fit from Equation 1. Inset: magnified view to show the break points (I_break_) where the cells transition from no firing or phasic firing to tonic firing. **(F)** Summary of I_break_ current levels for individual cells. *p < 0.05; ***p < 0.001.

Radiate and planar neurons showed similar resting membrane potentials (Figure 2A, radiate: −63.1 ± 1.5 mV, *n* = 27; planar: −63.0 ± 2.3 mV, *n* = 28; unpaired *t* test: *t*_(53)_ = 0.074, *p* = 0.941) and input resistances (Figure 2B; radiate: 74.1 ± 31.1 MΩ, *n* = 27; planar: 75.1 ± 31.0 MΩ, *n* = 28; Mann Whitney test: *p* = 0.762). However, radiate cells showed significantly faster membrane time constant (2.9 ± 1.1 ms, *n* = 27) than that of the planar cells (3.8 ± 1.7 ms, *n* = 28; Figure 2C; Mann Whitney test: *p* = 0.024).

We next compared the excitability of these two types of neurons, by measuring the threshold potential (*V*_threshold_) to current injections and the current vs. firing rate function with 100 ms long positive current injections (Figures 2D–F). As previously reported in planar neurons (Xie and Manis, 2013b), the threshold potential was measured as the membrane potential at which the second derivative was the maximum preceding the action potential (Figure 2D). Only the measurement at the first action potential was used from the trace with action potentials evoked by the minimum current injection level. On average, there was a trend for radiate neurons to have a higher (more depolarized) threshold potential than planar neurons in response to current injections (Figure 2D; radiate V_threshold_: −39.5 ± 4.8 mV, *n* = 27; planar V_threshold_: −41.7 ± 3.5 mV, *n* = 28; unpaired *t* test: *t*_(53)_ = 1.91, *p* = 0.062). Notably, in response to the threshold level of the current injection, most radiate neurons (59%, 16 out of 27 neurons) fired transiently with only one or a few action potentials that began within 30 ms after the onset of current injection (Figure 1A), whereas this pattern was less frequently seen in planar cells (7%, 2 out of 28 neurons). The rest of the planar neurons fired regular trains of action potentials at all current levels (Figures 1C,D). The probability of observing transient firing was significantly higher in the radiate than planar cell samples (Fisher’s exact test, *p* < 0.0001). Once regular firing commenced, the firing rates of the cells depended on the current level for both radiate and planar cells. As shown in Figures 1A–D, 2E, the spike count increased monotonically with increasing current in the majority of neurons (25 out of 27 radiate neurons, and 27 out of 28 planar neurons). In two radiate and one planar neurons, the spike count decreased for larger currents, presumably due to depolarization block. The current-spike count curves for cells not exhibiting depolarization block were individually fit with Eq. 1, and the fit parameters were compared between the two cell populations. The only parameter that was significantly different between the planar and radiate populations was the breakpoint for the current, *I*_break_, which is the current level where the cells transit from no firing or only transient firing to entering a limit cycle that produces regular trains of action potentials (*I*_break_ for planar: 94.8 ± 59.5 pA, radiate: 207.4 ± 127.9 pA; *t*_(35)_ = −3.80, *p* = 0.00067; Figure 2F). This result is consistent with the higher threshold for the radiate neurons (Figure 2D), but also indicates that once firing commences, the increase in rate with current (*I*_r_, planar: 614.6 ± 368.3, radiate: 554.4 ± 254.9; *t*_(48)_ = 0.67, *p* = 0.51), and the maximal firing rates (*F*_max_ for 100 ms steps, planar: 79.6 – 26.7, radiate: 86.5 ± 45.8; *t*_(39)_ = −0.62, *p* = 0.54) are similar in the two populations. Thus, radiate cells are less excitable near threshold than planar cells, in spite of having similar input resistances and resting potentials and maximal firing rates.

### sEPSC Events in Radiate Multipolar Neurons Are More Heterogeneous in Amplitude and Kinetics than in Planar Multipolar Neurons

**Figure 3:**
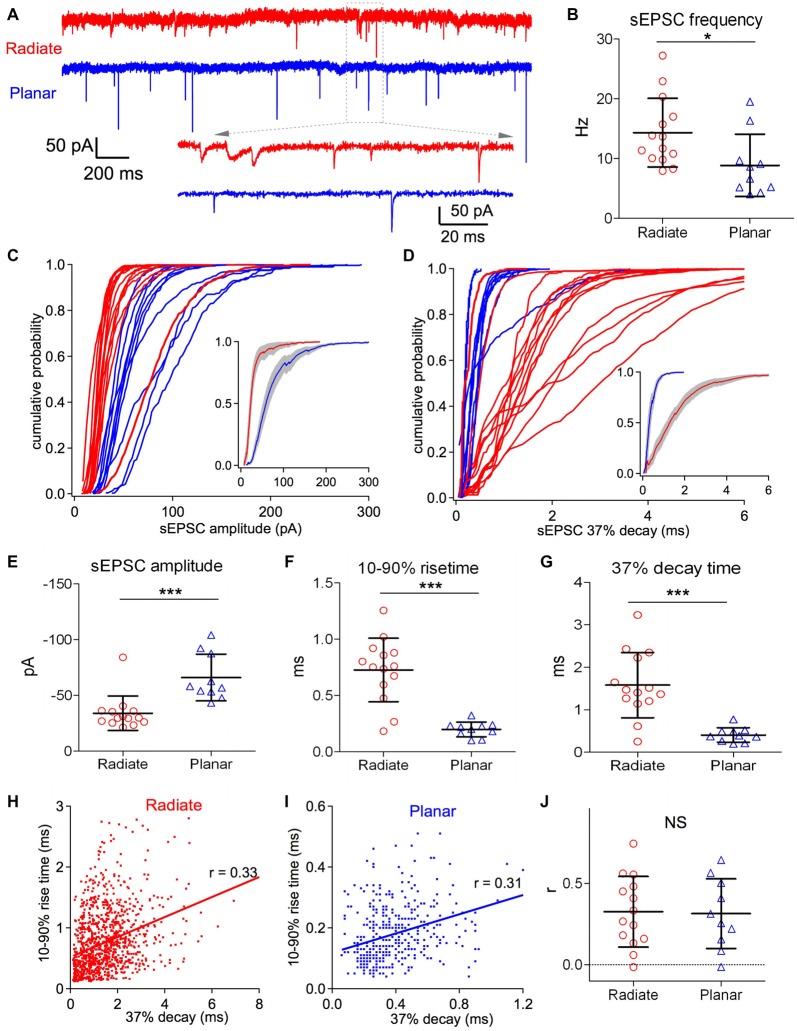
Radiate cells have more diverse spontaneous excitatory postsynaptic current (sEPSCs) with smaller amplitude and slower kinetics than planar cells. **(A)** Example traces of sEPSC events from a radiate (red) and a planar cell (blue). Inset: magnified view of individual sEPSC events. Notice the variations in both size and kinetics of sEPSC events in the radiate neuron. Color-coding applies throughout the figure. **(B)** Average sEPSC event frequency in both cell types. *p < 0.05. **(C)** Cumulative probability of the sEPSC event amplitudes for radiate and planar cells. Each curve represents an individual cell. Inset: average of the cumulative probability from all cells in each cell type; gray shaded areas represent the standard error of the mean. **(D)** Cumulative plots of the sEPSC 37% decay time. Inset: average of the cumulative plots from all cells in each cell type. **(E–G)** Summary plots of the average sEPSC amplitude **(E)**; average sEPSC 10%–90% rise time **(F)**; and average sEPSC 37% decay time **(G)**. ***p < 0.001. **(H,I)** Example plots of the 10%–90% rise time vs. peak to 37% decay time from individual sEPSC events of a radiate cell **(H)** and a planar cell **(I)**. Notice that the time scales in both the ordinate and the abscissa are different between **(H,I)**. Each dot represents an individual sEPSC event; line: linear regression; *r*: correlation coefficient. **(J)** Summary of all *r* values from both cell types. Each data point represents one cell. NS: not significant.

Both radiate and planar neurons receive excitatory synaptic inputs from the auditory nerve. However, despite the differences in their physiological responses to sound, it is unclear if there is any difference in the properties of their synaptic inputs from the auditory nerve. We evaluated the peak amplitude, rising and decay time courses, as well as synaptic dynamics of the excitatory synaptic inputs from the auditory nerve onto both radiate and planar neurons by assessing the sEPSCs recorded in voltage clamp. The sEPSCs in these two cell types differ in frequency, amplitude and time courses (Figure 3A). The frequency of sEPSC events was significantly higher in radiate cells than planar cells (Figure 3B; radiate sEPSC frequency: 14.3 ± 5.6 Hz, *n* = 14; planar: 8.9 ± 1.7 Hz, *n* = 10; unpaired *t* test: *t*_(22)_ = 2.38, *p* = 0.026). The cumulative plots in Figures 3C,D show that the sEPSC amplitudes and 37% decay times (see “Materials and Methods” section) are also differentially distributed between radiate and planar neurons. On average, radiate neurons had significantly smaller sEPSC amplitudes than planar neurons (Figure 3E; radiate: −34 ± 15 pA, *n* = 14; planar: −66 ± 21 pA, *n* = 10; Mann Whitney test: *p* = 0.0003). The sEPSCs in radiate cells were kinetically more diverse than those of planar neurons (Figures 3A,D), with radiate cells showing a significantly higher variance in both the 10%–90% rise time (F-test: *F*_(13,9)_ = 18.55, *p* < 0.0001; Figure 3F) and 37% decay time (F-test: *F*_(13,9)_ = 20.04, *p* < 0.0001; Figure 3G). The average 10%–90% rise time of sEPSC events was 0.73 ± 0.28 ms (*n* = 14) in radiate neurons, which was significantly slower than the 10%–90% rise time of 0.20 ± 0.07 ms (*n* = 10) in planar cells (Figure 3F; unpaired *t* test with Welch’s correction: *t*_(14)_ = 6.74, *p* < 0.0001). The average sEPSC 37% decay time was 1.58 ± 0.77 ms (*n* = 14) in radiate cells, whereas it was 0.41 ± 0.17 ms (*n* = 10) in planar cells (Figure 3G; unpaired *t* test with Welch’s correction: *t*_(14)_ = 5.53, *p* < 0.0001).

Assuming that all of the synapses give rise to conductances with similar time courses, the sEPSCs arising from synapses at scattered locations along dendrites are expected to show a correlation between the rise and decay times (Rall et al., 1992). We tested this prediction by examining the correlation between the 10%–90% rise time and the 37% decay time of individual sEPSCs. As shown in Figures 3H,I, the 10%–90% rise time and 37% decay time of individual sEPSC events were generally positively correlated. For 12 out of 14 radiate and 9 out of 10 planar neurons, the slope of the linear regression between 10%–90% rise time and 37% decay time of sEPSC events in each neuron was significantly different from 0 (F-tests: *p* < 0.05). The average correlation coefficient (r value) was 0.33 ± 0.22 (*n* = 14) in radiate neurons, and was not significantly different from the correlation (*r* = 0.31 ± 0.21, *n* = 10) in planar neurons (Figure 3J; unpaired *t* test: *t*_(22)_ = 0.13, *p* = 0.897). This result is consistent with the proposal that somatically-recorded sEPSCs in both radiate and planar neurons are shaped by dendritic filtering. The mostly smaller and slower sEPSC events observed in radiate cells (Figures 3C–G) may reflect the more extensive distribution of synaptic inputs along their longer dendrites (White et al., 1994), or might result from a difference in AMPA receptor subunit composition at the auditory nerve synapses.

Standard cable theory would predict that there should be a correlation between event amplitude and time course. Therefore, we also examined the correlation between peak amplitude and the 37% decay time of individual sEPSC events. In 5 out of 10 planar neurons, the slope of the linear regression between peak amplitude and 37% decay time was significantly different from 0 (F-tests: *p* < 0.01). The linear regression slopes in all these five neurons were negative, suggesting that sEPSC amplitude was negatively correlated with the 37% decay time in these neurons, as would be expected from the effects of dendritic filtering. The slopes of the linear regression in other five planar neurons were not significantly different from 0 (F-tests > 0.05). In comparison, the slope of linear regression between sEPSC amplitude and 37% decay time was significantly different from 0 (F-tests: *p* < 0.001) in 10 out of 14 radiate neurons. However, five of these radiate neurons showed negative correlation (negative slope), while, surprisingly, the other five cells showed a positive correlation (positive slope) between sEPSC amplitude and 37% decay time. No significant difference was found in the correlation coefficient (r value) between all radiate (*r* = − 0.068 ± 0.29; *n* = 14) and planar neurons (*r* = − 0.082 ± 0.16). While the results of negative correlation between sEPSC amplitude and 37% decay time can be well explained by the mechanism of dendritic filtering, the positive correlation in some radiate neurons requires an alternative explanation.

### Evoked EPSCs (eEPSCs)

**Figure 4:**
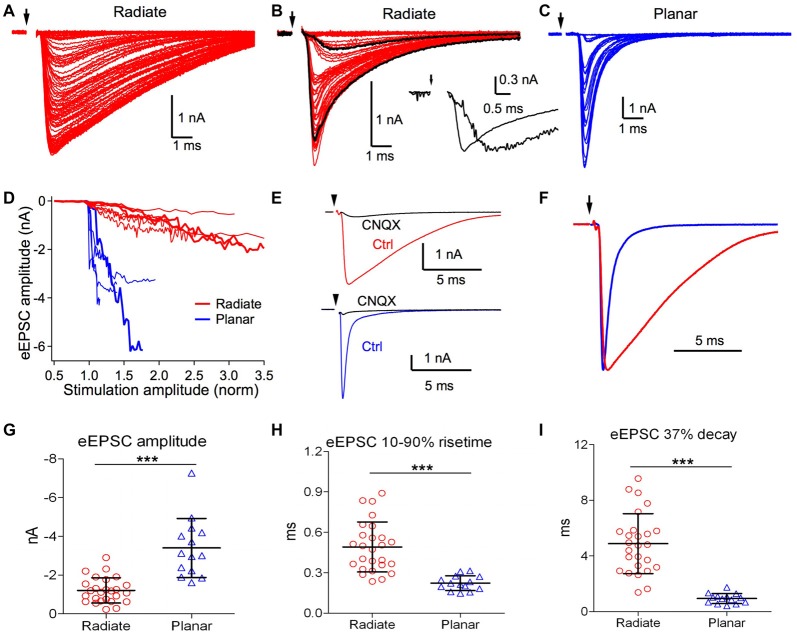
Radiate neurons receive weaker and slower evoked synaptic inputs than planar neurons. **(A,B)** Example eEPSCs of radiate neurons (red) with graded amplitudes driven by stimuli with progressively increased strength. Arrow: onset of electric stimulation. Notice in **(B)** that a fast eEPSC component was recruited beyond a certain stimulus strength that changed the shape of eEPSCs. Black traces in **(B)**, an example slow eEPSC evoked by weak stimulation and a fast eEPSC evoked by strong stimulation. Inset: the two black traces were normalized to the peak amplitude to show the temporal differences in eEPSCs driven by stimuli at different strength. **(C)** Example eEPSCs of a planar neuron (blue) driven by stimuli with progressively increased strength. The increase in eEPSC amplitude occurred over a narrow stimulus range, and grew in a stepwise fashion. Note the scale differences in eEPSC amplitudes among **(A–C)**. **(D)** Summary plot of eEPSC amplitudes vs. normalized stimulation amplitudes in radiate and planar neurons. Each line represents one individual neuron. Thick red lines mark the example radiate neurons in **(A,B)**, and the thick blue line marks the example planar neuron in **(C)**. The stimulation amplitude was normalized to the threshold stimulus current used in the individual neuron, which is 1.0 in the *x*-axis. **(E)** Example eEPSCs of a radiate and a planar neuron that were blocked by CNQX (cells held at −77 mV). **(F)** Comparison of the time course of eEPSCs (normalized to peak) from example radiate and planar neurons. **(G–I)** Summary of the eEPSC amplitude **(G)**, 10%–90% rise time **(H)**, and 37% decay time **(I)**. ***p < 0.001.

We next studied eEPSCs driven by electrical stimulation at the auditory nerve root. Responses were measured for a range of stimulus strengths from below threshold for evoking eEPSCs to the maximal eEPSC amplitude. As shown in Figure 4A, eEPSCs in radiate neurons showed numerous small increments in amplitude with increasing stimulation strength, suggesting recruitment of additional small synaptic inputs with stronger stimulation. The recruited synaptic inputs often varied in time course at different stimulation strength, as shown in an example radiate neuron in Figure 4B, such that stronger stimulation evoked faster eEPSCs that peaked over 1 ms earlier than eEPSCs driven by weaker stimulation. This variation in eEPSC time course is consistent with spatially distributed synaptic inputs along the extensive neural architecture of radiate neurons (Rall et al., 1992). In planar neurons, the increase in eEPSC amplitude often showed discrete steps (Figure 4C), consistent with previous measurements indicating that planar neurons receive a small number (~5) of auditory nerve inputs (Ferragamo et al., 1998; Cao and Oertel, 2010). The eEPSC amplitude in planar neurons grew rapidly with stimulus current before reaching their maximal amplitude, often saturating within 150% of the threshold stimulus current (Figure 4D). In contrast, eEPSCs in radiate neurons showed a much shallower growth in amplitude that continued beyond 300% of the threshold current (Figure 4D).

We further studied the properties of excitatory synaptic inputs to radiate and planar neurons using maximal eEPSCs. As shown in Figure 4E, eEPSCs were largely blocked by bath-application of 5 μM CNQX in both radiate neurons (83 ± 13%, *n* = 7) and planar neurons (89 ± 12%, *n* = 4; Mann Whitney test: *p* = 0.6485), suggesting that eEPSCs at −77 mV in both cell types are mediated by AMPA receptors. The strength and kinetics of the eEPSCs were strikingly different between radiate and planar neurons. As shown in Figures 4A–C,G, the maximal eEPSC amplitude in radiate neurons (−1.2 ± 0.7 nA; *n* = 26) was significantly smaller than planar neurons (−3.4 ± 1.5 nA; *n* = 14; unpaired *t* test: *t*_(38)_ = 6.38, *p* < 0.0001). Consistent with the sEPSCs, the kinetics of the eEPSCs in radiate neurons was also significantly slower than in planar neurons (Figure 4F). The 10%–90% rise time of eEPSCs in radiate neurons was significantly longer than in planar neurons (Figure 4H; radiate: 0.49 ± 0.19 ms, *n* = 26; planar: 0.22 ± 0.05 ms, *n* = 14; unpaired *t* test: *t*_(38)_ = 5.26, *p* < 0.0001); and the 37% decay time was slower (Figure 4I; radiate: 4.88 ± 2.16 ms, *n* = 26; planar: 0.94 ± 0.36 ms, *n* = 14; unpaired *t* test: *t*_(38)_ = 6.74, *p* < 0.0001).

In summary, the gradual and shallow growth of eEPSC amplitude in radiate neurons over a broad range of stimulus strength, as well as the significantly smaller eEPSC amplitude, suggest that radiate neurons receive weaker but more numerous synaptic inputs from the auditory nerve than planar neurons. The variable but generally slower eEPSC kinetics in radiate neurons suggests that these synaptic inputs are spatially more dispersed along their dendritic trees, and are consistent with these neurons receiving auditory nerve inputs that originate from a wide span of cochlear locations (Smith and Rhode, 1989; Palmer et al., 1996, 2003; Arnott et al., 2004). Alternatively, radiate neurons may express different glutamate receptor subunits with slower kinetics than those in planar neurons.

### Short-Term Synaptic Plasticity during Repetitive Stimulation

**Figure 5:**
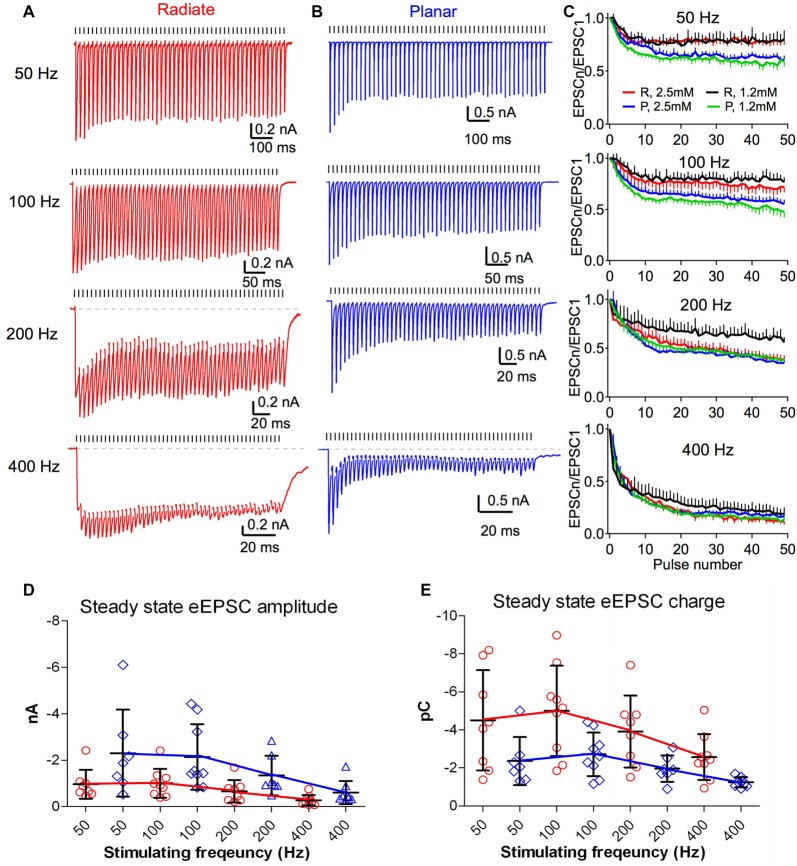
Short term dynamics of auditory nerve synaptic inputs differs between radiate and planar cells. **(A,B)** Example eEPSC responses of a radiate cell **(A)** and a planar cell **(B)** to stimulus trains at different frequencies. Tick marks above the traces indicate the timing of the stimulus pulses. Dashed lines in the 200 Hz and 400 Hz trains mark the baseline of the EPSC responses. Color-coding applies to the entire figure (red: radiate; blue: planar). **(C)** Summary of the synaptic depression during stimulus trains at 50, 100, 200 and 400 Hz. EPSC_n_/EPSC_1_: all eEPSC sizes were normalized to the amplitude of the first eEPSC. Color traces in each panel showed recordings made under artificial cerebrospinal fluid (ACSF) containing either 2.5 mM Ca^2+^ and 1.5 mM Mg^2+^ (red and blue) or 1.2 mM Ca^2+^ and 0.8 mM Mg^2+^ (black and green). R: radiate neurons; P: planar neurons. **(D)** Summary of the average steady-state eEPSC amplitudes during the last 40 pulses of the stimulus trains in both cell types. Note that the plots are staggered to show the individual cells. **(E)** Summary of the average steady-state eEPSC charge during the last 40 pulses of the stimulus trains. Each symbol in **(D,E)** represents an individual neuron.

The auditory nerve fibers can fire at rates >400 Hz with acoustic stimulation (Taberner and Liberman, 2005; Wen et al., 2009). During such repetitive activity in the presence of elevated extracellular calcium (2–2.5 mM), auditory nerve synapses onto bushy and planar multipolar neurons exhibit prominent short-term synaptic depression (Wang and Manis, 2008; Yang and Xu-Friedman, 2009, 2015; Cao and Oertel, 2010). However, the short-term synaptic plasticity of the auditory nerve inputs to radiate neurons has not been studied. As in previous experiments (Xie and Manis, 2013b), we evaluated synaptic dynamics of eEPSCs in radiate neurons, compared to planar neurons using 50-pulse trains of auditory nerve root stimulation at 50, 100, 200 and 400 Hz (Figure 5). eEPSCs were depressed during repetitive stimulation in both radiate and planar cells (Figures 5A,B). It is worth noting that the depression was much less in radiate neurons at low rates (50 and 100 Hz), although it was similar at high rates (200 and 400 Hz; Figure 5C). A two-way ANOVA revealed that the magnitude of synaptic depression was significantly different between the two cell types (*F*_(1,392)_ = 28.1, *p* < 0.0001), and not surprisingly, also among stimulus rates (*F*_(3,392)_ = 302.1, *p* < 0.0001). There was also a significant interaction between cell type and stimulus rate (*F*_(3,392)_ = 6.1, *p* = 0.0005). Bonferroni-corrected posttests showed that radiate neurons had significantly less depression than planar neurons during trains at 50 and 100 Hz (*p* < 0.0001 for both frequencies), but depressed to the same level at 200 and 400 Hz (*p* > 0.05 for both frequencies). To quantify the steady-state depression level, we calculated the average depression from the last 40 pulses of the stimulus train. Relative to the amplitude of the first eEPSC of the train, eEPSCs in radiate cells depressed to 78.6 ± 1.3% at 50 Hz (*n* = 8), 74.7 ± 2.5% at 100 Hz (*n* = 9), 48.6 ± 6.7% at 200 Hz (*n* = 8), and 19.0 ± 6.5% at 400 Hz (*n* = 9); whereas planar cells depressed to 64.7 ± 2.8% at 50 Hz (*n* = 7), 61.9 ± 3.2% at 100 Hz (*n* = 9), 43.3 ± 4.6% at 200 Hz (*n* = 7), and 20.8 ± 3.6% at 400 Hz (*n* = 8).

We also assessed the short-term synaptic plasticity in both radiate and planar neurons using an ACSF with physiological divalent ion concentrations (“Materials and Methods” section). Similar to eEPSC trains recorded in 2.5 mM external calcium, synaptic depression was also observed throughout the trains in 1.2 mM calcium at all stimulus rates in both cell types (Figure 5C; black and green lines). A two-way ANOVA revealed that the magnitude of synaptic depression was significantly different between two cell types (*F*_(1,392)_ = 176.0, *p* < 0.0001) and across stimulus rates (*F*_(3,392)_ = 337.2, *p* < 0.0001). There was a significant interaction between cell type and stimulus rate (*F*_(3,392)_ = 8.33, *p* < 0.0001). Bonferroni-corrected posttests showed that radiate neurons had significantly less depression than planar neurons at all stimulus rates (*p* < 0.0001 at 50, 100 and 200 Hz; *p* < 0.05 at 400 Hz). Relative to the first eEPSC of the train, the average depression of the last 40 pulses of the stimulus train in radiate neurons were 77.8 ± 1.7% at 50 Hz, 79.8 ± 1.8% at 100 Hz, 64.9 ± 3.9% at 200 Hz, and 26.4 ± 5.3% at 400 Hz (*n* = 5); whereas the average depression in planar cells were 60.5 ± 2.6% at 50 Hz, 56.3 ± 3.7% at 100 Hz, 46.9 ± 5.2% at 200 Hz, and 18.5 ± 4.7% at 400 Hz (*n* = 6).

As eEPSCs in radiate neurons have significantly slower kinetics (Figures 4F,H,I) than those in planar neurons, the effectiveness of synaptic inputs can be influenced more by temporal summation. This is visible for radiate neurons at 200 and 400 Hz in Figure 5A, and to a lesser extent, for planar neurons in Figure 5B. To quantify the effects of summation, we measured the average peak eEPSC amplitude (Figure 5D) and the eEPSC charge (Figure 5E) during the last 40 pulses of the EPSC trains, where the eEPSC amplitudes were at steady-state. The peak eEPSC amplitude was measured relative to the current level immediately preceding the measured EPSC to remove any baseline offset. A linear mixed-model was used to evaluate the EPSC amplitude, with stimulus rate and cell type as fixed effects. The steady-state eEPSC peak amplitude depended on both cell type (*F*_(1,15.9)_ = 5.23, *p* = 0.036; F-test with Kenward-Roger approximation) and stimulus rate (*F*_(3,44.8)_ = 6.51, *p* < 0.0001). There was no significant interaction (*F*_(3,41.7)_ = 2.31, *p* = 0.09). In spite of the weaker synaptic depression at low frequencies and similar depression at high frequencies, the radiate neurons showed overall smaller eEPSC peak amplitude during the steady-state of the train than that of the planar neurons (simultaneous tests for general linear hypotheses between cell types: 50 Hz: *t* = −2.90, *p* = 0.007; *t* = −2.56, *p* = 0.016 at 100 Hz; *p* ≥ = 0.15 for 200 and 400 Hz; *p* values adjusted for multiple comparisons by Holm’s method; Figure 5D). The average eEPSC charge (Figure 5E), measured as the integrated area under each eEPSC referenced to the baseline during the last 40 pulses of the EPSC trains, reflects the overall impact of individual events and temporal summation onto the target cell during a stimulus cycle. A linear mixed model revealed that the average EPSC charge transfer also depended on cell type (*F*_(1,15.9)_ = 7.46, *p* = 0.015) and stimulus rate (*F*_(3,44.5)_ = 17.4, *p* < 0.0001). There was no interaction between cell type and stimulus rate (*F*_(3,41.5)_ = 0.29, *p* = 0.84). In spite of the smaller eEPSC peak amplitude in radiate neurons throughout the trains, the average EPSC charge transfer was higher in radiate neurons than planar neurons. Post-tests revealed that the difference was significant at 100 and 200 Hz but not at 50 and 400 Hz (50 Hz: *t* = 2.68, *p* = 0.13; 100 Hz: *t* = 3.01, *p* = 0.006, 200 Hz: *t* = 2.442, *p* = 0.022; 400 Hz: *t* = 1.53, *p* = 0.14; Holm’s adjusted). Thus, during ongoing synaptic activity, the temporal summation of slower EPSCs can play an important role in the radiate multipolar neurons.

### Spike Rate and Timing in Response to Repetitive Auditory Nerve Stimulation

We next studied the spike rate and timing in radiate and planar neurons in response to trains of repetitive auditory nerve stimulation in current-clamp. Spikes evoked by the last 40 stimuli of the 50-pulse trains were used in order to assess the steady state responses.

**Figure 6:**
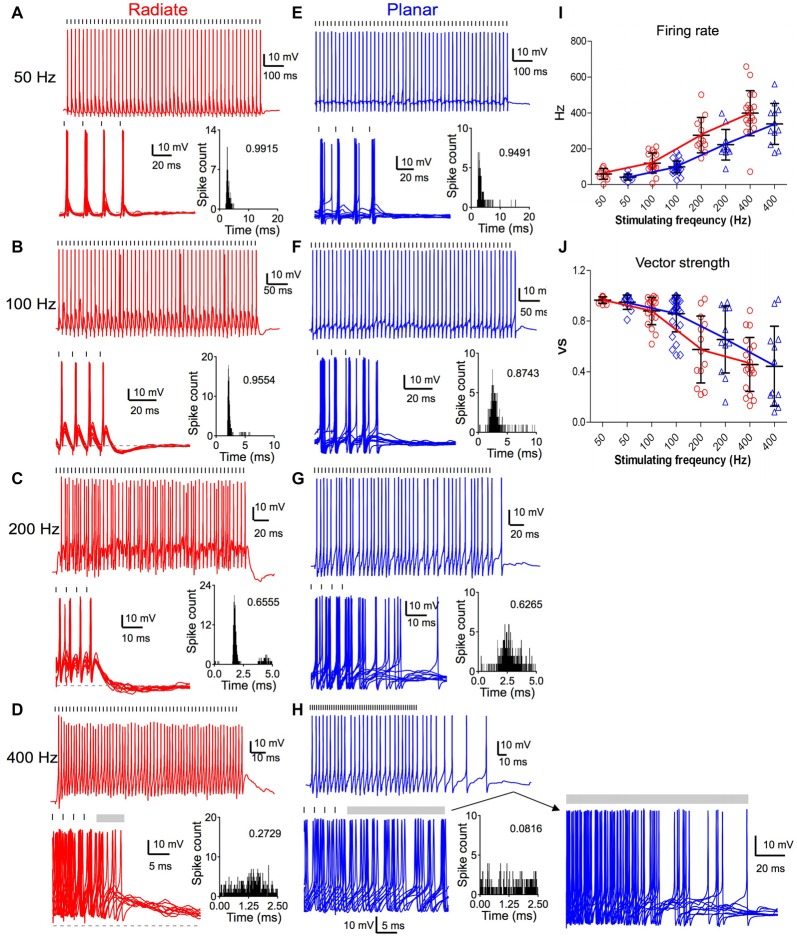
Comparison of suprathreshold spike entrainment between radiate and planar cells. **(A)** Example responses to 50 pulse stimulus trains at 50 Hz. Top panel: a single trace response to the stimulus train; bottom left panel: overlap of 10 traces to stimulus trains (only the last four stimuli of the trains are shown); gray dashed line marks the resting membrane potential; bottom right panel: overlapped peristimulus time histogram (PSTH) plot to show spike timing within the stimulus cycle; the number in the plot is the calculated vector strength. Tick marks above the traces indicate the timing of the stimulus pulses. **(B–D)** Example responses of the same radiate cell as in **(A)** to stimulus trains at 100 Hz **(B)**, 200 Hz **(C)** and 400 Hz **(D)**. Labels and layout are the same as in **(A)**. Gray bars after the stimulus trains mark the window of delayed spikes in **(D,H)**. **(E–H)** Example responses of a planar cell to stimulus trains at 50 Hz **(E)**, 100 Hz **(F)**, 200 Hz **(G)**, and 400 Hz **(H)**. Notice that the spikes of radiate and planar cells occur at consistent latency relative to the stimulus at 50 and 100 Hz, but increasing latency variability reduces the precision at 200 and 400 Hz. **(I)** Summary of the firing rates in response to trains of stimulations at different frequencies. **(J)** Summary of the vector strength in response to trains of stimulations at different frequencies.

Both radiate and planar neurons fired tonic trains of spikes in response to trains of auditory nerve stimulation. In response to 50 Hz trains, both cell types fired regularly with approximately a single spike per stimulus throughout the train (Figures 6A,E). The spikes showed little jitter in response to each stimulus, and the period histograms showed a single sharp peak. As the stimulating frequency was increased, the firing rate of both cell types increased (Figures 6A–I). However, the evoked spikes became temporally less precise relative to the onset of individual stimuli, as indicated by the dispersion of spike times within the stimulus period (Figures 6B–D,F–H). The firing rate of radiate neurons was 60 ± 30 Hz to 50 Hz stimulation (*n* = 11); 120 ± 55 Hz to 100 Hz stimulation (*n* = 19); 277 ± 99 Hz to 200 Hz stimulation (*n* = 13) and 399 ± 126 Hz to 400 Hz stimulation (*n* = 19; Figure 6I). The firing rate of planar cells was 43 ± 17 Hz to 50 Hz stimulation (*n* = 11); 99 ± 33 Hz to 100 Hz stimulation (*n* = 29); 223 ± 85 Hz to 200 Hz stimulation (*n* = 11) and 338 ± 114 Hz to 400 Hz stimulation (*n* = 12). A linear mixed model revealed that the firing rate was strongly dependent on stimulus frequency (*F*_(3,84.6)_ = 164.4, *p* < 0.0001), but was not dependent on the cell type (*F*_(1,42.8)_ = 3.56, *p* = 0.066; Figure 6I). Overall, the radiate neurons fired spikes at higher rates than planar neurons, although Bonferroni posttests did not reveal any significant difference at any individual stimulation rate except at 400 Hz (*p* > 0.12 for 50–200 Hz; *t* = −2.65, *p* = 0.0093 at 400 Hz; Holm’s adjusted). There was no interaction between cell type and stimulus frequency (*F*_(3,81.1)_ = 1.60, *p* = 0.19).

The temporal precision of spike timing through the train was quantified by measuring the vector strength (Goldberg and Brown, 1969). The average vector strength in radiate cells was 0.964 ± 0.026 (*n* = 11) at 50 Hz, 0.879 ± 0.108 (*n* = 19) at 100 Hz, 0.574 ± 0.264 (*n* = 13) at 200 Hz, and 0.457 ± 0.214 (*n* = 19) at 400 Hz; whereas in planar cells it was 0.948 ± 0.057 (*n* = 11), 0.858 ± 0.144 (*n* = 29), 0.654 ± 0.264 (*n* = 11), and 0.443 ± 0.316 (*n* = 12), respectively (Figure 6J). A linear mixed model revealed that the vector strength depended on stimulus frequency (*F*_(3,87.6)_ = 61.3, *p* < 0.0001), but not on cell type (*F*_(1,42.3)_ = 0.0026, *p* = 0.96). The vector strength decreased with increasing stimulus frequency. There was no significant interaction between stimulus frequency and cell type (*F*_(3,84.8)_ = 1.01, *p* = 0.39). Thus, the radiate and planar neurons respond to synchronous auditory nerve activity with comparable temporal precision (Figure 6J; *p* > 0.17 at all frequencies; Holm’s adjusted), in spite of the differences in the time course and convergence of their auditory nerve inputs. The temporal precision is preserved for low frequency stimulation in both cell types, but at higher afferent rates there appears to be a gradual transition to a spike rate representation (Figures 6I,J), consistent with the increased temporal summation of synaptic inputs.

Sensory information can be faithfully represented in neurons that fire reliably during the ongoing course of a stimulus, and promptly stop firing when the stimulus is off. However, we noted that this was not the case for all multipolar cells, especially after high frequency stimulation. Therefore, we analyzed the offset responses that followed the end of the excitatory stimulus trains. Radiate neurons promptly ceased firing upon the termination of stimulus trains at 50 Hz and 100 Hz, and only fired occasional delayed spikes after stimulus trains at 200 and 400 Hz (Figures 6C,D). On average, radiate neurons fired for 0.6 ± 1.1 ms (*n* = 13) immediately following 200 Hz trains and for 18.0 ± 26.9 ms (*n* = 19) after 400 Hz stimulus trains. At 50 and 100 Hz, the majority of the planar neurons also ceased firing immediately after the end of the stimulus train (Figure 6E). Only 3 out of 29 planar cells fired additional spikes following the termination of 100 Hz stimulus trains. One of these is shown in Figure 6F, where it fired a single delayed spike. However, when auditory nerve fibers (ANFs) were stimulated at 200 and 400 Hz, planar neurons fired delayed spikes well beyond the end of the stimulation (Figure 6H). The continued firing lasted for 8.9 ± 14.0 ms (*n* = 11) following 200 Hz trains and for 64.5 ± 52.0 ms (*n* = 12) following 400 Hz stimulus trains. A linear mixed model revealed a significant interaction between cell type and stimulus frequency (*F*_(1,87.4)_ = 7.18, *p* < 0.0001) for the log-transformed duration of delayed firing. Furthermore, the duration of the delayed firing was significantly dependent on both cell type (*F*_(1,38.1)_ = 13.2, *p* = 0.0008) and frequency (*F*_(1,94.4)_ = 64.0, *p* < 0.0001). Posttests confirm that radiate neurons promptly terminate their spiking at the end of repetitive stimulation compared to planar neurons at 200 and 400 Hz (200 Hz: *t* = 3.10, *p* = 0.0024; 400 Hz: *t* = 5.10, *p* < 0.0001; Holm’s adjusted), whereas no difference was seen for stimulation at 50 or 100 Hz (*p* > 0.5). A close examination of the traces (Figure 6H, lower right) indicates that this firing may be driven by a barrage of EPSPs that persist for tens of ms after the stimulus ends. Late EPSPs also are evident in the radiate cells following termination of the train (Figure 6D), although they appear less efficacious in supporting sustained firing. Similar delayed EPSPs following auditory nerve stimulation have been reported before in the planar stellate cell population (Ferragamo et al., 1998).

## Discussion

AVCN radiate neurons differ from planar neurons in their intrinsic excitability as well as the kinetics and dynamics of excitatory synaptic inputs from auditory nerve. First, we found that radiate neurons are less excitable than planar neurons, as they tend to have a higher current threshold for triggering action potentials, and have a faster membrane time constant. However, the two cell types have the same maximum firing rate for large current injections. Second, the radiate neurons receive numerous smaller, kinetically variable but slower excitatory synaptic inputs from the auditory nerve, consistent with a spatial dispersion of synaptic inputs along their dendritic arbors. Third, radiate neurons have a shallow growth of eEPSC amplitude in response to graded increases in stimulus current over a wide range, which suggests the integration of a larger number of inputs than seen in planar cells. Fourth, radiate neurons had a smaller maximal eEPSC amplitude. Fifth, radiate neurons show less synaptic depression at low rates and more temporal summation at high rates. The results suggest that radiate neurons have unique intrinsic and synaptic properties to support their physiological function as the “wide-band inhibitor” (Nelken and Young, 1994; Palmer et al., 2003; Arnott et al., 2004), by integrating a large number of auditory inputs over a broad frequency range and providing reliable and strong inhibition to target neurons.

### Intrinsic Excitability

Radiate neurons are associated with the O_c_ discharge pattern in response to tone bursts at their best frequency (Smith and Rhode, 1989; Winter and Palmer, 1995; Palmer et al., 2003; Arnott et al., 2004). This pattern consists of a brief burst of at least two reproducibly timed spikes, followed by desynchronized firing at a much lower rate. We found that, unlike the planar neurons, the radiate neurons had a tendency to fire phasically at the lowest current levels. This pattern is similar to the “Type I–II” discharge patterns in the models of Rothman and Manis (2003), which were created based on measurements of potassium currents in a population of VCN neurons. The Type I–II model included a small amount of the low-voltage activated potassium current, which serves to prevent the cell entering a repetitive firing regime during weak depolarization, but allows the cells to fire repetitively for stronger depolarization. The presence of a regime in the radiate multipolar cells where they fire transiently suggests that a weak low-voltage activated potassium conductance could contribute to their discharge pattern in response to acoustic stimuli. We note that this pattern was not seen in all radiate cells, which may be a methodological limitation of using relatively coarse (50 pA) current steps when constructing the I–V curves, or may represent diversity in the excitability of these cells. A more careful investigation of the firing patterns just at threshold, along with testing whether α-dendrotoxin-sensitive (Kv1 family) potassium channels are present, in comparison to the planar cells, might provide additional insights into the regulation of the discharge patterns of the radiate cells. It is also possible that the prominent I_h_ currents in the radiate cells could contribute to driving onset responses, because when the cells are depolarized, the portion of the I_h_ current that is open at rest will further reinforce the depolarization until the conductance decreases at the new depolarized voltage level.

The intrinsic excitability of radiate neurons seems unlikely to entirely account for their responses to acoustic stimuli. Inhibitory inputs, auditory nerve convergence and dendritic filtering are also likely to contribute to the onset chopper pattern. Onset chopper cells have weak inhibitory areas relative to the chop-T and chop-S populations (Rhode and Greenberg, 1994), which are associated with planar multipolar cells. Spontaneous IPSCs were seen in radiate neurons in this study (not shown). In two putative radiate cells, evoked inhibitory inputs from nearby sites in the AVCN were also seen using glutamate uncaging (Campagnola et al., 2014). The onset chopper pattern can also in principle be produced by convergence of auditory nerve fibers from a wide frequency region (Sumner et al., 2009), particularly if the inputs are subject to low-pass filtering associated with dendritic electrotonus. We found evidence for convergence and low-pass filtering when examining the excitatory synaptic currents in radiate neurons.

### EPSC Kinetics

There were significant differences in the variability of the rise and decay times of sEPSC and eEPSCs between the radiate and planar cells. The results may be explained by multiple distinct mechanisms: (1) a differential distribution of synaptic inputs and dendritic filtering; (2) a differential expression of glutamate receptor subunits; or (3) by variations in distance-dependent amplitudes of synaptic conductances or dendritic input resistance.

First, if we assume that these excitatory synaptic inputs all arise from a common source (the auditory nerve synapses), and that they are associated with a single pattern of glutamate receptor subunit expression in both cell types, so that the time course of the conductance events are the same at the synaptic site, then the best explanation for our result is that synaptic inputs to the planar cells are electrotonically relatively close to the soma, whereas those in the radiate cells are located across a range of electrotonic distances. The EPSC measurements were made under conditions where the ability to voltage clamp synaptic events that are located even a short distance away from the soma is compromised by the cable properties of the dendrites (Spruston et al., 1993; Williams and Mitchell, 2008). As a result, electrotonically distant synaptic conductances are both poorly clamped, and have a distorted time course with slower rise and decay times (Rall et al., 1992). Although the variability in the ability to clamp synaptic currents at various electrotonic distances certainly could contribute to the differences in the average sEPSC amplitude that we observed, whether this issue is the sole factor or whether the synaptic conductances are themselves heterogeneous cannot be resolved with this approach. Of particular relevance to this explanation, radiate neurons have fairly extensive dendritic trees. The only previous estimates of the electrotonic structure of VCN multipolar cells were made in guinea pigs (White et al., 1994). In that study, cells were found to have an effective (collapsed cylinder) electrotonic length of either ~1 or 1.5, depending on the ratio of the somatic to the dendritic resistivity; however, no attempt was made to differentiate between radiate and planar neurons. Regardless, this is certainly long enough that the dendrites will provide significant low-pass filtering of distal synaptic inputs. In cat, the radiate multipolar cells receive strong somatic input (Cant, 1981), and the cells can phase lock nearly as well as auditory nerve fibers (Smith and Rhode, 1989), suggesting that inputs proximal to the spike initiation zone play an important role in spike timing. In comparison, the chop-S and chop-T (associated with planar) neurons are generally poorer phase lockers (Blackburn and Sachs, 1990), consistent with dendritic filtering of synaptic events resulting from a principally dendritic innervation pattern (White et al., 1994). The wide range of sEPSC rise and decay times in mouse radiate multipolar cells suggests that excitatory synapses have a broad spatial distribution, including both somatic and dendritic sites. This is also supported by the shape of the eEPSCs, which decay with a time course that appears to have multiple components (Figure 4B), as would be expected for spatially distributed synaptic conductances.

Second, radiate neurons may express different AMPA receptor subunits that produce slower conductance changes than those in planar neurons, which can contribute to the observed differences in EPSC kinetics. Glutamate receptors are known to be differentially distributed in different areas of the cochlear nucleus as well as different compartments of individual neurons (Rubio and Wenthold, 1997; Petralia et al., 2000; Gardner et al., 2001). EPSC kinetics also differ amongst the different principal cells of the cochlear nucleus (Gardner et al., 1999), although in that study radiate multipolar cells were not explicitly characterized. It is possible that radiate neurons express GluR2 subunits, resulting in slower kinetics, whereas planar neurons express receptors biased towards GluR3/4 subunits, with fast kinetics. Alternatively, if both cells express GluR3/4 subunits, the radiate neurons may have GluR3/4 subunits in slower “flip” variant, while planar neurons have the subunits in faster “flop” variant (Sommer et al., 1990; Mosbacher et al., 1994).

Third, the synaptic inputs at different positions along the dendrite may encounter different input resistances as a result of the taper of dendrite diameter and distance-dependent ion channel densities (Winters et al., 2017), which would lead to a different pattern of somatic voltage changes with distance than expected solely from electrotonic decay. A related possibility is the presence of distance-dependent scaling of the synaptic conductances (Stricker et al., 1996; Magee and Cook, 2000), such that the more distal dendritic inputs have larger conductances. Regardless of the mechanism, it is clear that synaptic integration in the two multipolar cell types is quite different, and is influenced by the dendritic locations of synapses and the electrotonic structure of the cell.

### Auditory Nerve Convergence

Our measurements suggest, not surprisingly, greater convergence from auditory nerve fibers onto radiate cells than onto planar cells. Previous measurements have suggested that 5–7 ANFs (Ferragamo et al., 1998; Cao and Oertel, 2010) converge onto individual planar cells. Based on quantal size (66 pA), the mean maximal EPSC amplitude (3.4 nA), and an average of 5.5 converging ANFs, we can estimate that action potentials in single auditory nerve fibers may release nine quanta per stimulus onto planar multipolar cells. This corresponds to a mean single-fiber EPSC of ~0.59 nA, which even taking into account the differences in driving force between experiments, is at the top end of the range reported by Cao and Oertel (2010) for planar multipolar cells. With an average resting release probability of 0.4 under the standard recording conditions (Xie and Manis, 2013b, Table 1), each ANF would be predicted to have as many as 20 presynaptic sites onto an individual planar multipolar neuron. The presence of discrete steps in previous studies (Ferragamo et al., 1998; Cao and Oertel, 2010), which were occasionally visible in our limited data set, together with a typical CV of 0.3 for quantal size, provides supporting evidence for such a large number of active sites per ANF onto the target cell; otherwise individual increments in EPSC amplitude with recruitment of individual presynaptic fibers would not be resolvable. Although this estimated convergence onto planar multipolar neurons is greater than that estimated onto bushy cells (2–4; Cant and Morest, 1979; Oertel, 1983; Cao and Oertel, 2010), it is not exceptionally high. In contrast, the number of release sites is only about 20% of the estimate of 80–100 onto mouse bushy cells (Nicol and Walmsley, 2002; Wang and Manis, 2008).

Because we were unable to resolve the steps associated with recruitment of individual ANFs in the EPSCs onto radiate multipolar cells, we cannot estimate AN convergence and quantal content. However, we can provide upper and lower bounds. If we assume that each ANF that innervates a radiate neuron makes a single synaptic contact, and assume the same resting release probability as in planar cells (0.4), then the maximal EPSC amplitude of 1.2 nA and a quantal size of 0.034 nA suggest an upper bound of 88 ANFs. If the release at each site is governed by the same statistics as for the planar multipolar cells, then the lower bound must be at least twice as large as in planar cells, or ~11 ANFs. Otherwise we would expect to be able to resolve the recruitment of individual fibers as the stimulation intensity is increased.

### Temporal Precision

The ability of radiate and planar multipolar cells to follow repetitive stimulation of the auditory nerve was similar. The cells could entrain on a cycle-by-cycle basis at 50 and 100 Hz, but began to fire less synchronously at 200 and 400 Hz, although the overall firing rate continued to increase. The loss of temporal precision arose from accumulated depolarization during the stimulus train. The accumulated depolarization includes activation of slower NMDA receptors (Cao and Oertel, 2010; Xie and Manis, 2013b), as well as (in the radiate cells) the slower time course of the eEPSC. The relationship between these responses and the acoustic responses of multipolar cells is not straightforward. In general, the planar multipolar cells do not phase lock as well as bushy cells to tones above about 1 kHz (Blackburn and Sachs, 1989). The radiate cells (O_c_ units) in chinchilla VCN have been found to have the poorest synchronized rates for low frequency modulated stimuli (Shofner et al., 1996), although they show high phase-locking using traditional measures, possibly due to relatively sparse spiking. However, using different measures, Rhode and Greenberg (1994) found that the O_c_ units in cat VCN had the best encoding for phase locking to SAM stimuli, showing a low-pass behavior in their modulation transfer functions with a corner frequency of at least 1 kHz for some cells. In contrast, the other chopper classes that are associated with planar cells had peaked (band-pass) functions, with a corner frequency near 500 Hz. The ability of the radiate cells to generate temporally precise responses to such inputs would seem to be compromised by their dendritic filtering. However, such precision may result from a stronger excitatory somatic input, and their broader modulation transfer functions may be related to the greater AN convergence across frequency.

### Delayed Firing after High Rate Activity

Compared to radiate neurons, planar neurons showed prominent delayed firing long after the termination of high rate stimulus inputs from the auditory nerve (Figures 6D,H). Planar neurons have axon collaterals within the VCN, which could provide feed-forward excitation to other planar multipolar cells (and other cell types; reviewed by Oertel et al., 2011), and there is some prior evidence for this (Ferragamo et al., 1998). In particular, with high rate stimulation, the activation of many cells in the nucleus could generate sufficient recurrent excitation to support sustained firing in target planar neurons beyond the termination of auditory nerve stimulation. It is also possible that inhibition onto or amongst radiate cells helps terminate spiking, although there is no direct evidence for a difference in inhibitory inputs between radiate and planar neurons, nor were IPSPs consistently observed following the stimulus trains. The radiate cells may also not be as densely innervated by the collaterals of the planar cells as other planar cells. The rapid termination of spiking in radiate cells might also be due to their faster membrane time constant, their higher current threshold for spiking (Figures 2C–F), the engagement of different activity-dependent ion channels (such as a Ca^2+^- or Na^+^-dependent K channels), or differential expression of synaptic receptors whose activation would be rate dependent (NMDA, metabotropic glutamate) between the two cell types.

In summary, we find that radiate and planar multipolar cells of the cochlear nuclei differ quantitatively and qualitatively in terms of intrinsic excitability, convergence and the strength and dynamics of auditory nerve synaptic input. These features likely contribute to their different responses to acoustic stimuli, and the roles that they play in auditory information processing.

## Author Contributions

RX and PBM designed the research, analyzed the data and wrote the manuscript. RX performed electrophysiological experiments.

## Conflict of Interest Statement

The authors declare that the research was conducted in the absence of any commercial or financial relationships that could be construed as a potential conflict of interest.

## Footnotes

^1^www.gimp.org

^2^http://scipy.org

## References

[B1] Adams J. C. (1983). Multipolar cells in the ventral cochlear nucleus project to the dorsal cochlear nucleus and the inferior colliculus. Neurosci. Lett. 37, 205–208. 10.1016/0304-3940(83)90431-7 6888799

[B2] Arnott R. H. Wallace M. N. Shackleton T. M. Palmer A. R. (2004). Onset neurones in the anteroventral cochlear nucleus project to the dorsal cochlear nucleus. J. Assoc. Res. Otolaryngol. 5, 153–170. 10.1007/s10162-003-4036-8 15357418 PMC2538402

[B3] Blackburn C. C. Sachs M. B. (1989). Classification of unit types in the anteroventral cochlear nucleus: PST histograms and regularity analysis. J. Neurophysiol. 62, 1303–1329. 2600627 10.1152/jn.1989.62.6.1303

[B4] Blackburn C. C. Sachs M. B. (1990). The representations of the steady-state vowel sound /e/ in the discharge patterns of cat anteroventral cochlear nucleus neurons. J. Neurophysiol. 63, 1191–1212. 2358869 10.1152/jn.1990.63.5.1191

[B5] Campagnola L. Kratz M. B. Manis P. B. (2014). ACQ4: an open-source software platform for data acquisition and analysis in neurophysiology research. Front. Neuroinform. 8:3. 10.3389/fninf.2014.00003 24523692 PMC3906568

[B6] Cant N. B. (1981). The fine structure of two types of stellate cells in the anterior division of the anteroventral cochlear nucleus of the cat. Neuroscience 6, 2643–2655. 10.1016/0306-4522(81)90109-3 7322355

[B7] Cant N. B. (1982). Identification of cell types in the anteroventral cochlear nucleus that project to the inferior colliculus. Neurosci. Lett. 32, 241–246. 10.1016/0304-3940(82)90300-7 7177487

[B8] Cant N. B. Morest D. K. (1979). Organization of the neurons in the anterior division of the anteroventral cochlear nucleus of the cat. Light-microscopic observations. Neuroscience 4, 1909–1923. 10.1016/0306-4522(79)90065-4 530438

[B9] Cao X. J. Oertel D. (2010). Auditory nerve fibers excite targets through synapses that vary in convergence, strength and short-term plasticity. J. Neurophysiol. 104, 2308–2320. 10.1152/jn.00451.2010 20739600 PMC3350034

[B10] Clements J. D. Bekkers J. M. (1997). Detection of spontaneous synaptic events with an optimally scaled template. Biophys. J. 73, 220–229. 10.1016/S0006-3495(97)78062-7 9199786 PMC1180923

[B13] Doucet J. R. Ross A. T. Gillespie M. B. Ryugo D. K. (1999). Glycine immunoreactivity of multipolar neurons in the ventral cochlear nucleus which project to the dorsal cochlear nucleus. J. Comp. Neurol. 408, 515–531. 10.1002/(sici)1096-9861(19990614)408:4<515::aid-cne6>3.3.co;2-f 10340502

[B11] Doucet J. R. Ryugo D. K. (1997). Projections from the ventral cochlear nucleus to the dorsal cochlear nucleus in rats. J. Comp. Neurol. 385, 245–264. 10.1002/(sici)1096-9861(19970825)385:2<245::aid-cne5>3.3.co;2-u 9268126

[B12] Doucet J. R. Ryugo D. K. (2006). Structural and functional classes of multipolar cells in the ventral cochlear nucleus. Anat. Rec. A Discov. Mol. Cell. Evol. Biol. 288, 331–344. 10.1002/ar.a.20294 16550550 PMC2566305

[B14] Ferragamo M. J. Golding N. L. Oertel D. (1998). Synaptic inputs to stellate cells in the ventral cochlear nucleus. J. Neurophysiol. 79, 51–63. 9425176 10.1152/jn.1998.79.1.51

[B15] Frisina R. D. Smith R. L. Chamberlain S. C. (1990). Encoding of amplitude modulation in the gerbil cochlear nucleus: I. A hierarchy of enhancement. Hear. Res. 44, 99–122. 10.1016/0378-5955(90)90074-y 2329098

[B16] Fujino K. Oertel D. (2001). Cholinergic modulation of stellate cells in the mammalian ventral cochlear nucleus. J. Neurosci. 21, 7372–7383. 11549747 10.1523/JNEUROSCI.21-18-07372.2001 PMC6763002

[B17] Gardner S. M. Trussell L. O. Oertel D. (1999). Time course and permeation of synaptic AMPA receptors in cochlear nuclear neurons correlate with input. J. Neurosci. 19, 8721–8729. 10516291 10.1523/JNEUROSCI.19-20-08721.1999 PMC6782765

[B18] Gardner S. M. Trussell L. O. Oertel D. (2001). Correlation of AMPA receptor subunit composition with synaptic input in the mammalian cochlear nuclei. J. Neurosci. 21, 7428–7437. 11549753 10.1523/JNEUROSCI.21-18-07428.2001 PMC6763000

[B19] Goldberg J. M. Brown P. B. (1969). Response of binaural neurons of dog superior olivary complex to dichotic tonal stimuli: some physiological mechanisms of sound localization. J. Neurophysiol. 32, 613–636. 5810617 10.1152/jn.1969.32.4.613

[B20] Jiang D. Palmer A. R. Winter I. M. (1996). Frequency extent of two-tone facilitation in onset units in the ventral cochlear nucleus. J. Neurophysiol. 75, 380–395. 8822565 10.1152/jn.1996.75.1.380

[B21] Lauer A. M. Connelly C. J. Graham H. Ryugo D. K. (2013). Morphological characterization of bushy cells and their inputs in the laboratory mouse (Mus musculus) anteroventral cochlear nucleus. PLoS One 8:e73308. 10.1371/journal.pone.0073308 23991186 PMC3753269

[B22] Magee J. C. Cook E. P. (2000). Somatic EPSP amplitude is independent of synapse location in hippocampal pyramidal neurons. Nat. Neurosci. 3, 895–903. 10.1038/78800 10966620

[B23] McGinley M. J. Oertel D. (2006). Rate thresholds determine the precision of temporal integration in principal cells of the ventral cochlear nucleus. Hear. Res. 216–217, 52–63. 10.1016/j.heares.2006.02.006 16647828

[B24] Mellott J. G. Motts S. D. Schofield B. R. (2011). Multiple origins of cholinergic innervation of the cochlear nucleus. Neuroscience 180, 138–147. 10.1016/j.neuroscience.2011.02.010 21320579 PMC3070814

[B25] Mosbacher J. Schoepfer R. Monyer H. Burnashev N. Seeburg P. H. Ruppersberg J. P. (1994). A molecular determinant for submillisecond desensitization in glutamate receptors. Science 266, 1059–1062. 10.1126/science.7973663 7973663

[B26] Muniak M. A. Rivas A. Montey K. L. May B. J. Francis H. W. Ryugo D. K. (2013). 3D model of frequency representation in the cochlear nucleus of the CBA/J mouse. J. Comp. Neurol. 521, 1510–1532. 10.1002/cne.23238 23047723 PMC3992438

[B27] Needham K. Paolini A. G. (2003). Fast inhibition underlies the transmission of auditory information between cochlear nuclei. J. Neurosci. 23, 6357–6361. 12867521 10.1523/JNEUROSCI.23-15-06357.2003 PMC6740532

[B28] Needham K. Paolini A. G. (2006). Neural timing, inhibition and the nature of stellate cell interaction in the ventral cochlear nucleus. Hear. Res. 216–217, 31–42. 10.1016/j.heares.2006.01.016 16554129

[B29] Nelken I. Young E. D. (1994). Two separate inhibitory mechanisms shape the responses of dorsal cochlear nucleus type IV units to narrowband wideband stimuli. J. Neurophysiol. 71, 2446–2462. 7931527 10.1152/jn.1994.71.6.2446

[B30] Nicol M. J. Walmsley B. (2002). Ultrastructural basis of synaptic transmission between endbulbs of Held and bushy cells in the rat cochlear nucleus. J. Physiol. 539, 713–723. 10.1113/jphysiol.2001.012972 11897843 PMC2290185

[B31] Oertel D. (1983). Synaptic responses and electrical properties of cells in brain slices of the mouse anteroventral cochlear nucleus. J. Neurosci. 3, 2043–2053. 6619923 10.1523/JNEUROSCI.03-10-02043.1983 PMC6564561

[B32] Oertel D. Wright S. Cao X. J. Ferragamo M. Bal R. (2011). The multiple functions of T stellate/multipolar/chopper cells in the ventral cochlear nucleus. Hear. Res. 276, 61–69. 10.1016/j.heares.2010.10.018 21056098 PMC3078527

[B33] Oertel D. Wu S. H. Garb M. W. Dizack C. (1990). Morphology and physiology of cells in slice preparations of the posteroventral cochlear nucleus of mice. J. Comp. Neurol. 295, 136–154. 10.1002/cne.902950112 2341631

[B34] Palmer A. R. Jiang D. Marshall D. H. (1996). Responses of ventral cochlear nucleus onset and chopper units as a function of signal bandwidth. J. Neurophysiol. 75, 780–794. 8714652 10.1152/jn.1996.75.2.780

[B35] Palmer A. R. Wallace M. N. Arnott R. H. Shackleton T. M. (2003). Morphology of physiologically characterised ventral cochlear nucleus stellate cells. Exp. Brain Res. 153, 418–426. 10.1007/s00221-003-1602-6 12955380

[B37] Paolini A. G. Clarey J. C. Needham K. Clark G. M. (2005). Balanced inhibition and excitation underlies spike firing regularity in ventral cochlear nucleus chopper neurons. Eur. J. Neurosci. 21, 1236–1248. 10.1111/j.1460-9568.2005.03958.x 15813933

[B36] Paolini A. G. Clark G. M. (1999). Intracellular responses of onset chopper neurons in the ventral cochlear nucleus to tones: evidence for dual-component processing. J. Neurophysiol. 81, 2347–2359. 10322071 10.1152/jn.1999.81.5.2347

[B38] Petralia R. S. Rubio M. E. Wang Y. X. Wenthold R. J. (2000). Differential distribution of glutamate receptors in the cochlear nuclei. Hear. Res. 147, 59–69. 10.1016/s0378-5955(00)00120-9 10962173

[B39] Pressnitzer D. Meddis R. Delahaye R. Winter I. M. (2001). Physiological correlates of comodulation masking release in the mammalian ventral cochlear nucleus. J. Neurosci. 21, 6377–6386. 11487661 10.1523/JNEUROSCI.21-16-06377.2001 PMC6763188

[B40] Rall W. Burke R. E. Holmes W. R. Jack J. J. Redman S. J. Segev I. (1992). Matching dendritic neuron models to experimental data. Physiol. Rev. 72, S159–S186. 1438585 10.1152/physrev.1992.72.suppl_4.S159

[B41] Rhode W. S. Greenberg S. (1994). Encoding of amplitude modulation in the cochlear nucleus of the cat. J. Neurophysiol. 71, 1797–1825. 8064349 10.1152/jn.1994.71.5.1797

[B43] Rhode W. S. Oertel D. Smith P. H. (1983). Physiological response properties of cells labeled intracellularly with horseradish peroxidase in cat ventral cochlear nucleus. J. Comp. Neurol. 213, 448–463. 10.1002/cne.902130408 6300200

[B42] Rhode W. S. Smith P. H. (1986). Encoding timing and intensity in the ventral cochlear nucleus of the cat. J. Neurophysiol. 56, 261–286. 3760921 10.1152/jn.1986.56.2.261

[B44] Rich A. W. Xie R. Manis P. B. (2010). Hearing loss alters quantal release at cochlear nucleus stellate cells. Laryngoscope 120, 2047–2053. 10.1002/lary.21106 20824788 PMC3091373

[B45] Rodrigues A. R. Oertel D. (2006). Hyperpolarization-activated currents regulate excitability in stellate cells of the mammalian ventral cochlear nucleus. J. Neurophysiol. 95, 76–87. 10.1152/jn.00624.2005 16192334

[B46] Rothman J. S. Manis P. B. (2003). The roles potassium currents play in regulating the electrical activity of ventral cochlear nucleus neurons. J. Neurophysiol. 89, 3097–3113. 10.1152/jn.00127.2002 12783953

[B47] Rouiller E. M. Ryugo D. K. (1984). Intracellular marking of physiologically characterized cells in the ventral cochlear nucleus of the cat. J. Comp. Neurol. 225, 167–186. 10.1002/cne.902250203 6327782

[B48] Rubio M. E. Wenthold R. J. (1997). Glutamate receptors are selectively targeted to postsynaptic sites in neurons. Neuron 18, 939–950. 10.1016/s0896-6273(00)80333-5 9208861

[B49] Shannon R. V. Zeng F. G. Kamath V. Wygonski J. Ekelid M. (1995). Speech recognition with primarily temporal cues. Science 270, 303–304. 10.1126/science.270.5234.303 7569981

[B50] Sherriff F. E. Henderson Z. (1994). Cholinergic neurons in the ventral trapezoid nucleus project to the cochlear nuclei in the rat. Neuroscience 58, 627–633. 10.1016/0306-4522(94)90086-8 7513389

[B51] Shofner W. P. Sheft S. Guzman S. J. (1996). Responses of ventral cochlear nucleus units in the chinchilla to amplitude modulation by low-frequency, two-tone complexes. J. Acoust. Soc. Am. 99, 3592–3605. 10.1121/1.414957 8655791

[B53] Smith P. H. Massie A. Joris P. X. (2005). Acoustic stria: anatomy of physiologically characterized cells and their axonal projection patterns. J. Comp. Neurol. 482, 349–371. 10.1002/cne.20407 15669051

[B52] Smith P. H. Rhode W. S. (1989). Structural and functional properties distinguish two types of multipolar cells in the ventral cochlear nucleus. J. Comp. Neurol. 282, 595–616. 10.1002/cne.902820410 2723154

[B54] Sommer B. Keinänen K. Verdoorn T. A. Wisden W. Burnashev N. Herb A.. (1990). Flip and flop: a cell-specific functional switch in glutamate-operated channels of the CNS. Science 249, 1580–1585. 10.1126/science.1699275 1699275

[B55] Spruston N. Jaffe D. B. Williams S. H. Johnston D. (1993). Voltage- and space-clamp errors associated with the measurement of electrotonically remote synaptic events. J. Neurophysiol. 70, 781–802. 8410172 10.1152/jn.1993.70.2.781

[B56] Stricker C. Field A. C. Redman S. J. (1996). Statistical analysis of amplitude fluctuations in EPSCs evoked in rat CA1 pyramidal neurones *in vitro*. J. Physiol. 490, 419–441. 10.1113/jphysiol.1996.sp021155 8821140 PMC1158680

[B57] Sumner C. J. Meddis R. Winter I. M. (2009). The role of auditory nerve innervation and dendritic filtering in shaping onset responses in the ventral cochlear nucleus. Brain Res. 1247, 221–234. 10.1016/j.brainres.2008.09.054 18848923 PMC2653631

[B58] Swaminathan J. Heinz M. G. (2012). Psychophysiological analyses demonstrate the importance of neural envelope coding for speech perception in noise. J. Neurosci. 32, 1747–1756. 10.1523/JNEUROSCI.4493-11.2012 22302814 PMC3297360

[B59] Taberner A. M. Liberman M. C. (2005). Response properties of single auditory nerve fibers in the mouse. J. Neurophysiol. 93, 557–569. 10.1152/jn.00574.2004 15456804

[B60] Tolbert L. P. Morest D. K. Yurgelun-Todd D. A. (1982). The neuronal architecture of the anteroventral cochlear nucleus of the cat in the region of the cochlear nerve root: horseradish peroxidase labelling of identified cell types. Neuroscience 7, 3031–3052. 10.1016/0306-4522(82)90228-7 6298659

[B61] Wang Y. Manis P. B. (2008). Short-term synaptic depression and recovery at the mature mammalian endbulb of Held synapse in mice. J. Neurophysiol. 100, 1255–1264. 10.1152/jn.90715.2008 18632895 PMC2544465

[B62] Webster D. B. Trune D. R. (1982). Cochlear nuclear complex of mice. Am. J. Anat. 163, 103–130. 10.1002/aja.1001630202 7072613

[B63] Wen B. Wang G. I. Dean I. Delgutte B. (2009). Dynamic range adaptation to sound level statistics in the auditory nerve. J. Neurosci. 29, 13797–13808. 10.1523/JNEUROSCI.5610-08.2009 19889991 PMC2774902

[B64] Wenthold R. J. (1987). Evidence for a glycinergic pathway connecting the two cochlear nuclei: an immunocytochemical and retrograde transport study. Brain Res. 415, 183–187. 10.1016/0006-8993(87)90285-x 3304530

[B65] White J. A. Young E. D. Manis P. B. (1994). The electrotonic structure of regular-spiking neurons in the ventral cochlear nucleus may determine their response properties. J. Neurophysiol. 71, 1774–1786. 8064348 10.1152/jn.1994.71.5.1774

[B66] Wickesberg R. E. Whitlon D. Oertel D. (1994). *In vitro* modulation of somatic glycine-like immunoreactivity in presumed glycinergic neurons. J. Comp. Neurol. 339, 311–327. 10.1002/cne.903390302 8132865

[B67] Williams S. R. Mitchell S. J. (2008). Direct measurement of somatic voltage clamp errors in central neurons. Nat. Neurosci. 11, 790–798. 10.1038/nn.2137 18552844

[B68] Winter I. M. Palmer A. R. (1995). Level dependence of cochlear nucleus onset unit responses and facilitation by second tones or broadband noise. J. Neurophysiol. 73, 141–159. 7714560 10.1152/jn.1995.73.1.141

[B69] Winters B. D. Jin S. X. Ledford K. R. Golding N. L. (2017). Amplitude normalization of dendritic epsps at the soma of binaural coincidence detector neurons of the medial superior olive. J. Neurosci. 37, 3138–3149. 10.1523/JNEUROSCI.3110-16.2017 28213442 PMC5373109

[B70] Wu S. H. Oertel D. (1984). Intracellular injection with horseradish peroxidase of physiologically characterized stellate and bushy cells in slices of mouse anteroventral cochlear nucleus. J. Neurosci. 4, 1577–1588. 6726347 10.1523/JNEUROSCI.04-06-01577.1984 PMC6564985

[B71] Xie R. (2016). Transmission of auditory sensory information decreases in rate and temporal precision at the endbulb of Held synapse during age-related hearing loss. J. Neurophysiol. 116, 2695–2705. 10.1152/jn.00472.2016 27683884 PMC5133313

[B72] Xie R. Manis P. B. (2013a). Glycinergic synaptic transmission in the cochlear nucleus of mice with normal hearing and age-related hearing loss. J. Neurophysiol. 110, 1848–1859. 10.1152/jn.00151.2013 23904491 PMC3798943

[B73] Xie R. Manis P. B. (2013b). Target-specific IPSC kinetics promote temporal processing in auditory parallel pathways. J. Neurosci. 33, 1598–1614. 10.1523/JNEUROSCI.2541-12.2013 23345233 PMC3737999

[B74] Xie R. Manis P. B. (2014). GABAergic and glycinergic inhibitory synaptic transmission in the ventral cochlear nucleus studied in VGAT channelrhodopsin-2 mice. Front. Neural Circuits 8:84. 10.3389/fncir.2014.00084 25104925 PMC4109614

[B75] Xie R. Manis P. B. (2017). Synaptic transmission at the endbulb of Held deteriorates during age-related hearing loss. J. Physiol. 595, 919–934. 10.1113/JP272683 27618790 PMC5285728

[B76] Yang H. Xu-Friedman M. A. (2009). Impact of synaptic depression on spike timing at the endbulb of Held. J. Neurophysiol. 102, 1699–1710. 10.1152/jn.00072.2009 19587324 PMC2894642

[B77] Yang H. Xu-Friedman M. A. (2015). Skipped-stimulus approach reveals that short-term plasticity dominates synaptic strength during ongoing activity. J. Neurosci. 35, 8297–8307. 10.1523/JNEUROSCI.4299-14.2015 26019343 PMC4444548

